# Comparative analysis of pomological and phytochemical characteristics in white‐ and red‐fleshed pitaya ( *Hylocereus* spp.), with molecular docking insights into key bioactive compounds

**DOI:** 10.1002/jsfa.70322

**Published:** 2025-11-19

**Authors:** Kerem Mertoglu, Annik Fischer, Sina Zargarchi, Melekber Sulusoglu Durul, Magdalena Köpsel, Erdi Can Aytar, Ibrahim Bulduk, Barıs Kaki, Tuba Esatbeyoglu

**Affiliations:** ^1^ Department of Horticulture, Faculty of Agriculture Uşak University Uşak Türkiye; ^2^ Department of Molecular Food Chemistry and Food Development, Institute of Food and One Health Gottfried Wilhelm Leibniz University Hannover Hannover Germany; ^3^ Department of Horticulture, Faculty of Agriculture Kocaeli University Kocaeli Türkiye; ^4^ Department of Chemical Engineering Afyon Kocatepe University Afyon Türkiye; ^5^ Department of Animal Science, Faculty of Agriculture Uşak University Uşak Türkiye

**Keywords:** antioxidant activity, phenolics, organic acids, *in silico*, multivariate analysis

## Abstract

**BACKGROUND:**

Cacti, including pitaya (*Hylocereus* spp.), are rich in antioxidants that will undoubtedly gain importance under ongoing climate change as water resources decline. Yet the molecular basis linking composition to antioxidant function remains incompletely defined. We compared white‐fleshed *H. undatus* and red‐fleshed *H. polyrhizus* across physicochemical traits, integrating correlation, principal component analysis, and molecular docking to a human iron‐regulatory protein (IRP1).

**RESULTS:**

White‐fleshed fruits were larger and heavier (length 103.4 mm; width 60.2 mm; weight 204.7 g) than red‐fleshed (71.9 mm; 54.5 mm; 126.3 g). Conversely, red‐fleshed fruits showed higher total soluble solids (13.47 *vs.* 9.60 °Brix), total phenolics (379.7 *vs.* 183.0 mg L^−1^), total flavonoids (303.7 *vs.* 147.3 mg L^−1^), and antioxidant activity (52.3% *vs.* 30.0%). Organic acids and phenolics differed by species (e.g., higher citric acid in red; higher malic acid in white). Correlations indicated that greater soluble sugars were associated with higher phenolic accumulation, consistent with the elevated antioxidant capacity of red‐fleshed fruit. The first two principal components explained 83.3% of the total variance (PC1 = 68.8%, PC2 = 14.5%) and separated samples along size/°Brix *versus* phenolic/ antioxidant axes. Docking predicted favorable binding of major acids and phenolics to IRP1, suggesting plausible antioxidant mechanisms.

**CONCLUSION:**

Findings support species‐specific use, with red‐fleshed *H. polyrhizus* serving as a nutrient‐dense source of antioxidant compounds, while white‐fleshed *H. undatus* offers advantageous pomological traits. Integrating compositional profiling with multivariate analysis and docking provides mechanistic context and practical implications for breeding, climate‐resilient cultivation, and industrial applications. © 2025 The Author(s). *Journal of the Science of Food and Agriculture* published by John Wiley & Sons Ltd on behalf of Society of Chemical Industry.

## INTRODUCTION

2023 was the warmest year after the first temperature record in 1850.^1^ High temperature increases stress levels and cause a decrease in water resources and an increase in agricultural salinity.^2^, ^3^ The combined effects of these stressors lead to the inactivation of key enzymes, denaturation, and precipitation of proteins, membrane integrity disruption, protein synthesis inhibition, oxidative stress, membrane damage, lipid peroxidation, pigment bleaching, and degradation of DNA strands in plants.^4^, ^5^ The damage leads to permanent cessation, depriving plants of their regular life cycle. In regions where these biotic stresses are predominant, it is necessary to assess the potential of cactus species bearing edible, nutritious fruits, like pitayas.

Pitaya (*Hylocereus* spp.) is from tropical and subtropical regions and belongs to the cactus family. The fruit has numerous health‐promoting attributes due to its diverse composition of bioactive compounds, including betalains, polyphenols, phenolic acids, flavonoids, fatty acids, terpenes, and sterols, along with essential vitamins and vital minerals. It also provides proteins, fats, carbohydrates, dietary fiber, phytoalbumins, and carotenoids, making pitaya a highly valuable dietary component.^6^, ^7^ This rich nutritional profile underlies a broad spectrum of biological activities, with studies demonstrating antioxidant, anti‐inflammatory, antimicrobial, and anticancer properties.^8^ These effects are mediated through complementary mechanisms. Phenolic acids and flavonoids neutralize free radicals and chelate transition metals, which alleviates oxidative stress and protects cellular integrity.^4^ Betalains contribute by reducing the production of pro‐inflammatory cytokines, thereby attenuating inflammation. Organic acids help regulate acidity and suppress microbial proliferation, while flavonoids promote apoptosis and induce cell cycle arrest in malignant cells.^8^ Taken together, these mechanisms clarify how pitaya's chemical composition results in measurable antioxidant and protective outcomes that support immune, cardiovascular, gastrointestinal, and metabolic health, as well as skin function, diabetes, obesity, and certain cancers, underscoring its potential as a functional food for disease prevention.^9^ Additionally, pitaya peels are rich in pectin, and its seed contains over 50% of the unsaturated fatty acids, including linoleic and linolenic acids, which are important for functional food properties.^10^, ^11^ As a functional species, pitaya stands out not only for its fresh consumption but also for its expanding industrial applications, which are reflected in the rising production volume and cultivated area.^12^, ^13^

The important traits that determine the significance of pitaya are largely shaped by its genetic background. Therefore, investigating how different pitaya species perform in various ecologies is crucial. A review article revealed pitaya's nutritional, ornamental, medicinal, and industrial values, and the importance of understanding its genetic resources and biotechnological tools used to enhance pitaya germplasm.^7^ Also, some other researchers provide insights into the species’ sensorial, morphological, and nutritional differences.^14^, ^15^ However, the number of studies in which the characteristics are diversified and thoroughly investigated is limited. Furthermore, although numerous studies have reported the nutritional composition and general antioxidant capacity of pitaya, detailed insights into the molecular mechanisms underlying these effects are still limited. Specifically, the structural interactions of major organic acids and phenolic compounds with human proteins involved in antioxidant defense have not been fully characterized. To address this gap, the present study employed molecular docking (MD) to evaluate the binding potential of pitaya's primary bioactive compounds with a key regulatory protein. MD is a computational technique that explores possible conformations of molecules within protein binding regions and applies scoring functions to identify the most favorable configuration at the target site.^16^ This complementary approach provides novel insights into how pitaya's characteristic compounds may contribute to antioxidant functionality at the molecular level, thereby linking compositional data with mechanistic understanding.

Therefore, the research focuses were (i) to analyze how variations in fruit flesh color influence physicochemical properties across different pitaya species, (ii) to determine the physicochemical characteristics of species, (iii) to explore the interactions between the studied traits using Pearson correlation analysis to guide farming practices, (iv) to apply principal component analysis to identify patterns of variation for provide essential insights for breeding efforts, and (v) to analyze the protein interactions of key compounds in pitaya, presenting as an initial study.

## MATERIAL AND METHODS

### Plant materials

The study utilized two distinct species – *H. undatus* (Hu) and *H. polyrhizus* (Hp) – from the *Hylocereus* genus. Fruits were collected from 3‐year‐old plants planted at 3.5 × 3.0 m intervals. Drip irrigation was used, and all routine cultural practices were applied. The plants were cultivated in Muğla, a region in Southwest Türkiye characterized by a subtropical climate. Fully ripened fruits from the ‘Siam Red’ (Hp, red peel and red flesh) and ‘Vietnam White’ (Hu, red peel and white flesh) pitaya varieties were utilized in the study.

### Determination of pomological characteristics, soluble solid content, and pH

The dimensions of each fruit at harvest were measured by its weight (g), length (mm), and equatorial diameter (mm). A digital caliper was used to record the fruit sizes (VWR‐6, Milan, Italy), while the fruit weight, pulp weight, and peel weight were recorded using an electronic balance (CPA 16001S, Sartorius, Göttingen, Germany). Peel thickness (mm) was also recorded using the digital vernier caliper at three fruit locations (at the top, middle, and bottom), and the average of these measurements was calculated according to Esquivel *et al*.^9^ A digital fruit hardness tester determined fruit flesh firmness. The fruits were punctured at two places at the center and periphery. The arithmetic mean of these values was calculated, and the results were given as kg cm^−2^.^17^ Fruits were sliced, and pulp was removed to calculate the flesh/whole fruit ratio based on their ratios to each other; results were expressed as a percentage (%).^18^ Then, the pulps (approximately one‐third of each) were pressed using a juice dispenser (Arzum AR1060, Istanbul, Türkiye), and the juice was obtained by filtering through coarse filter paper. The total soluble solids (TSS) in the juice samples were determined with a digital refractometer (Atago PR‐32, Tokyo, Japan), and the outcomes were expressed in °Brix.^19^ The juice pH was evaluated using a pH meter (Hach Co., Loveland, CO, USA).^20^

### Preparation of fruit extracts

To prepare extracts for analysis, 50 g of each replicate (from a mix of 10 fruits per replicate) was homogenized using 100 mL of 80% acetone mixed with 0.2% formic acid. The mixture was subjected to centrifugation at 20 000 × *g* for 20 min at a temperature of 4 °C. The resulting extracts were utilized for the analysis of phytochemicals. For high‐performance liquid chromatographic (HPLC) analysis, a 50 g sample was blended with 50 mL deionized water and shaken for 30 min at a controlled speed, and centrifuged at 5000 × *g* for 10 min at 4 °C; the supernatant was filtered through a 0.45 μm membrane.^21^

### Spectrophotometric analysis

#### Total phenolic content

The total phenolic content (TPC) was assessed using a modified Folin–Ciocâlteu method.^22^ In summary, 20 μL pitaya juice methanolic extracts, positive control ((+)‐catechin), calibration standards (100–500 mg L^−1^), and blank (bidistilled water) were placed in a 96‐well plate. Then, Folin–Ciocâlteu reagent, at a volume of 100 μL, was incorporated, followed by 100 μL sodium carbonate solution after 5 min of incubation. The final mixture is stored at ambient temperature (25 °C) for 2 h to allow hue emergence. The absorbance was measured at 765 nm using a microplate reader (Infinite M200, Tecan, Männedorf, Switzerland). The calibration curve was constructed using gallic acid as an external standard, and the results were reported as mg gallic acid equivalents L^−1^.

#### Total flavonoid content

The aluminum chloride colorimetric method, with modifications based on Nurcholis *et al*.,^23^ was employed for the analysis. A 96‐well plate was prepared in technical triplicate by adding 50 μL of the sample, rutin hydrate as the positive control, a 0.5 mg mL^−1^ quercetin stock solution as the calibration standard, and ethanol as the blank. Next, 130 μL ethanol was added to each well, followed by 20 μL of a 1:1 (v/v) mixture of 10% aluminum chloride solution and 1 mol L^−1^ sodium acetate.^23^ The plate was shaken for 12 s, and absorbance readings were taken at 415 nm after 40 min using a Infinite M200 spectrophotometer (Tecan, Männedorf, Switzerland) and results were expressed as mg quercetin L^−1^.

#### DPPH assay

The antioxidant activity of pitaya extracts was assessed following a modified version of the Brand‐Williams method.^24^ Briefly, 100 μL of a 40 μg mL^−1^ methanolic pitaya extract was dispensed into 96‐well plates, followed by the addition of 100 μL of a 300 μmol L^−1^ 2,2‐diphenyl‐1‐picrylhydrazyl (DPPH) solution. The mixture was incubated for 30 min, and absorbance was measured at 515 nm using an Infinite M200 spectrophotometer (Tecan). Antioxidant activity was quantified in Trolox equivalents, with untreated pitaya extract serving as the control and a 70:30 methanol–water /v/v) mixture used as the blank. Results were reported as a percentage, using ascorbic acid (500 mg citric acid mL^−1^) as the standard reference.

### Quantification of organic acids and phenolic compounds by HPLC–ultraviolet detection

An ultraviolet detector, quaternary pump, autosampler, and ChemStation software were included in the Agilent 1260 HPLC system (Agilent Technologies, Santa Clara, CA, USA) to quantify organic and phenolic acids. Organic acid separation was achieved on an ACE‐C18 column (4 mm × 150 mm, 5 μm; Hichrom Ltd, Theale, UK). The mobile phase was a 10 mmol L^−1^ aqueous potassium phosphate solution that was provided at a flow rate of 1 mL min^−1^ and had been adjusted to pH 2.2 with orthophosphoric acid. 20 μL of the sample was injected, and the detection wavelength of ascorbic acid was set at 245 nm, whereas the other organic acids were set at 210 nm.^25^

Phenolic compounds were assessed utilizing the identical column. Phase A (ultrapure water with 0.1% acetic acid) and Phase B (acetonitrile with 0.1% acetic acid) made up the mobile phase, which flowed at 1.0 mL min^−1^. The following was the gradient elution program: 8–10% B for 0–3.25 min; 10–12% B for 3.25–8 min; 12–25% B for 8–15 min; 25–30% B for 15–15.8 min; 30–90% B for 15.8–25 min; 90–100% B for 25–25.4 min; and 100% B for 25.4–30 min. A 10 μL injection was used, and the column temperature was maintained at 25 °C. The following detection wavelengths were chosen based on maximal absorption: caffeic and chlorogenic acids at 330 nm; vanillic acid at 225 nm; *p*‐coumaric acid at 305 nm; and syringic, protocatechuic, and gallic acids at 280 nm.^26^

### Quantification of sugars by HPLC–refractive index detection

Sugars, namely, sucrose, glucose, and fructose, were calculated using an Agilent HPLC 1200 Series system implemented with a G1312A binary pump, G7162A refractive index (RI) detector, and G1329A autosampler. Sugars were separated on a Lichrospher NH_2_ column (250 mm × 4.6 mm, 5 μm; CS Chromatographie Service, Langerwehe, Germany) with an NH_2_ pre‐column. The mobile phase consisted of a water and acetonitrile mixture (25:75, v/v) at a 1.0 mL min^−1^ flow rate. The column oven and RI detector temperatures were maintained at 35 °C. A 20 μL sample volume was injected. The samples were centrifuged for 10 min at 14 500 rpm after dilution with ultrapure water at 1/5 and 1/10 (v/v) ratios (Pico 21, Thermo Scientific, Darmstadt, Germany). Ultrapure water was used to dissolve all of the standards (glucose, fructose, and sucrose). The calibration standards ranged from 2.0 to 10.0 mg mL^−1^, as did the stock solutions (10 mg mL^−1^). Every result was expressed as g 100 mL^−1^.^27^

### Molecular docking studies

Molecular structures were created using Chem‐Draw Ultra 18.0 software (Cambridge Soft, Cambridge, MA, USA). The lowest energy conformations were generated with Chem 3D 18.0 and saved in Mol2 file format. Enzyme structures were retrieved from the Protein Data Bank (PDB). Small molecule ligands were docked into a specific pocket in the monoclinic crystal structure of human cytosolic aconitase (IRP1) (PDB ID: 2B3Y, resolution 1.85 Å) and preserved in PDB format. Interaction studies between molecules and enzymes were conducted using AutoDock Vina 1.5.7 software (Molecular Graphics Laboratory, Scripps Research Institute, La Jolla, CA, USA), and binding energies (kcal mol^−1^) were calculated.^28^ Visualizations of 2D and 3D structures were produced using BIOVIA Discovery Studio Visualizer (DS4.5, Accelrys, Inc., San Diego, CA, USA).^29^

### Statistical analysis

A randomized plot experimental layout was used, with five repetitions (plants). For pomological traits, measurements were carried out on 10 fruits from each repetition, collected from different areas of the plant to ensure uniform representation without overlap. For chemical analysis, the same 10 fruits were homogenized, and 50 g of subsamples was taken from these homogenates as a repetition for subsequent determinations. The statistical significance of differences between species was determined using a *t*‐test, and results were considered significant at *p* < 0.05. Pearson correlation analysis was performed to examine the relations among the traits investigated, and the results are presented together with the Pearson correlation coefficients. In addition, all parameters were subjected to principal component analysis (PCA) to identify patterns of variation and associations among traits. Both correlation and PCA analyses were conducted using R software (version 4.1.2, Vienna, Austria), and the PCA results were visualized using scatter plots of the first principal components.^30^, ^31^

## RESULTS AND DISCUSSION

Understanding the pomological and biochemical profiles is crucial for any improvement in breeding efficiency, fruit quality, and aspects of cultivation. This comparative study also established that white‐fleshed pitaya cultivars are superior in size parameters, while red‐fleshed ones were superior in phenolics, flavonoids, antioxidants, and soluble solids. Such differences in characteristics ensure a focused farming methodology by pointing toward different varieties with specific production requirements and commercial opportunities. Red‐ and white‐fleshed pitaya varieties differ in pomological traits. The whole fruit dimension of red‐fleshed pitaya is 54.51 mm wide and 71.86 mm long, with a weight of 126.30 g. White‐fleshed pitaya is slightly bigger – 60.22 mm wide and 103.40 mm long – and has a weight of 204.68 g. The fruit flesh dimensions have different widths, lengths, and weights between the two varieties. Red‐fleshed pitaya has a lower value than white‐fleshed (Table 1). The size and weight of the whole fruit and pulp are positively correlated and grow simultaneously in pitaya.^20^ Additionally, consistent with earlier ones, white‐fleshed pitaya varieties generally stand out regarding their pomology.^15^, ^32^ However, fruit flesh hardiness (around 3.10 kg cm^−2^) and flesh/whole fruit (72%) traits were found to be unimportant among varieties.

**Table 1:** Physicochemical characteristics of *Hylocereus undatus* (white) and *Hylocereus polyrhizus* (red).

	Red‐fleshed	White‐fleshed	*P*‐values	CV (%)
Whole fruit width (mm)	54.51 ± 2.82*	60.22 ± 0.77	0.028	6.34
Whole fruit length (mm)	71.86 ± 4.20**	103.40 ± 9.05	0.005	20.99
Whole fruit weight (g)	126.30 ± 21.4**	204.68 ± 8.73	0.004	27.40
Fruit flesh width (mm)	51.27 ± 3.99ns	55.81 ± 5.54	0.314	9.31
Fruit flesh length (mm)	57.82 ± 8.23*	84.14 ± 5.97	0.011	22.24
Fruit flesh weight (g)	91.50 ± 22.3*	146.30 ± 11.6	0.020	28.57
Fruit flesh firmness (kg cm^−2^)	3.13 ± 0.67ns	3.08 ± 0.51	0.922	17.04
Flesh/whole fruit (%)	71.83 ± 5.25ns	71.67 ± 6.03	0.973	7.05
Peel thickness (mm)	2.19 ± 0.14ns	2.53 ± 1.01	0.596	28.53
Total phenolic content (mg L^−1^)	379.67 ± 8.62***	183.00 ± 9.54	0.000	38.40
Total flavonoid content (mg L^−1^)	303.67 ± 6.43***	147.33 ± 8.02	0.000	38.08
Antioxidant activity (%)	52.33 ± 3.51**	30.00 ± 2.65	0.001	30.47
Soluble solid content (°Brix)	13.47 ± 0.31***	9.60 ± 0.46	0.000	18.61
pH	4.85 ± 0.03ns	4.78 ± 0.04	0.05	0.95

Asterisks indicate statistical difference between species at *p < 0.05, **p < 0.01, and ***p < 0.001; ns, non‐significant; CV, coefficient of variation.

Breaking down the biochemical composition of red‐ and white‐fleshed pitaya shows differences in antioxidant activity, soluble solids, TPC, and total flavonoid content (TFC). Red‐fleshed pitaya has higher TPC (379.67 mg L^−1^) and TFC (303.67 mg L^−1^) than white‐fleshed pitaya (183.00 and 147.33 mg L^−1^, respectively). Phenolics, especially flavonoids, reduce oxidative stress by acting as free radical scavengers, protecting cellular structures from oxidative damage. Additionally, they may influence key signaling pathways and enzymatic activities that contribute to the antioxidant defense system.^33^, ^34^ Red‐fleshed pitaya has better antioxidant activity (52.33%) than white‐fleshed pitaya (30.00%). However, the pH was insignificant, and both had a similar pH of around 4.80.

Analyzing organic acids, phenolic compounds, and sugar profiles provides further insights into the chemical composition of red‐ and white‐fleshed pitaya varieties (Table 2). Researchers identified malic acid to be the primary organic acid in pitaya.^19^, ^35^ The hierarchy of organic acids was consistent across varieties, with malic acid as the predominant, followed by citric acid, tartaric acid, oxalic acid, and ascorbic acid (Table 2). Organic acids play a crucial role in shaping taste – a key factor in consumer preference – as their degradation greatly contributes to the reduction of flavor.^36^ In addition to improving flavor, these acids keep the fruits' pH low, suppressing the development of spoilage‐causing microorganisms.^37^ Thus, maintaining the levels of organic acids is essential for ensuring the quality of fruits. Among the varieties, white‐fleshed reveals notable levels of malic acid (7.33 mg mL^−1^), tartaric acid (0.37 mg mL^−1^), and oxalic acid (0.47 mg mL^−1^), while citric acid (1.41 mg mL^−1^) was higher in red‐fleshed, and ascorbic acid was stable in both species, with 0.12 mg mL^−1^. In line with our results, white‐fleshed fruits differ with higher levels of malic acid (11.07 mg mL^−1^) and lower levels of citric acid (0.14 mg mL^−1^), compared to red‐fleshed fruits, which contain malic acid and citric acid, respectively, at 7.23 and 1.06 mg mL^−1^.^38^

**Table 2:** Organic acids, phenolic compounds, and sugar profile of *Hylocereus undatus* (white) and *Hylocereus polyrhizus* (red).

	Red‐fleshed	White‐fleshed	P‐value	CV (%)
Malic acid (mg mL^−1^)	6.20 ± 0.27*	7.33 ± 0.35	0.011	10.05
Citric acid (mg mL^−1^)	1.41 ± 0.08**	0.83 ± 0.07	0.001	29.08
Tartaric acid (mg mL^−1^)	0.25 ± 0.05*	0.37 ± 0.04	0.033	25.31
Oxalic acid (mg mL^−1^)	0.33 ± 0.04**	0.47 ± 0.02	0.004	20.62
Ascorbic acid (mg mL^−1^)	0.12 ± 0.02ns	0.12 ± 0.03	1.000	2.00
Chlorogenic acid (mg L^−1^)	22.37 ± 1.17**	36.87 ± 2.54	0.001	27.47
Ferulic acid (mg L^−1^)	7.97 ± 0.59**	5.95 ± 0.28	0.006	16.94
Gallic acid (mg L^−1^)	7.65 ± 0.25 ns	7.22 ± 0.22	0.577	2.90
*p*‐Coumaric (mg L^−1^)	2.23 ± 0.15ns	2.27 ± 0.16	0.802	6.13
Protocatechuic acid (mg L^−1^)	3.19 ± 0.13ns	3.06 ± 0.16	0.349	4.74
Caffeic acid (mg L^−1^)	1.08 ± 0.09ns	1.13 ± 0.10	0.604	7.95
Ellagic acid (mg L^−1^)	13.00 ± 0.76**	10.17 ± 0.60	0.007	14.40
Quercetin (mg L^−1^)	2.33 ± 0.32**	0.95 ± 0.14	0.002	48.23
Glucose (g 100 mL^−1^)	8.44 ± 0.11***	5.07 ± 0.49	0.000	27.68
Fructose (g 100 mL^−1^)	1.54 ± 0.07**	2.38 ± 0.16	0.001	23.96
Saccharose (g 100 mL^−1^)	0.81 ± 0.03**	0.56 ± 0.05	0.001	20.42
Glucose/Fructose	5.47 ± 0.18***	2.13 ± 0.07	0.000	48.23

Asterisks indicate statistical difference between species at *p < 0.05, **p < 0.01, and ***p< 0.001; ns, non‐significant; CV, coefficient of variation.

Three of the four phenolics, which were statistically significant, namely ellagic acid (13.00 mg L^−1^), ferulic acid (7.97 mg L^−1^), and quercetin (2.33 mg L^−1^), were in higher concentration in red‐fleshed fruits. These results indicate that red‐fleshed fruits possess a high concentration of phenolic compounds, crucial for their antioxidant capacity, color, and flavor through participation in many physiological processes. Chlorogenic acid, which stood out among the phenolic compounds investigated, in agreement with Tang *et al*.,^39^ varied from 22.37 mg L^−1^ (red‐fleshed) to 36.87 mg L^−1^ (white‐fleshed). Chlorogenic acid, which forms the majority of phenolic cycles in fruits, gives rise to more than 40 phenolic compounds – a number notably higher than that of other phenolics.^40^

Anthocyanins' precursor substrates are soluble sugars, which also function as signaling molecules in the phenylpropanoid pathway.^41^ Furthermore, fruits' perceived sweetness is influenced by their sugar content and composition. Regardless of the species, sugars remained consistent, with glucose being the most prevalent, followed by fructose and saccharose, respectively.^34^, ^42^ Elevated glucose (8.44 g 100 mL^−1^) and saccharose (0.81 g 100 mL^−1^) in red‐fleshed fruits are significantly higher than that in white‐fleshed fruits, whereas glucose (5.07 g 100 mL^−1^) and fructose (0.56 g 100 mL^−1^), at lower amounts, indicate a less pronounced sweetness profile. Previous studies reported that red‐fleshed pitaya varieties exhibit high levels of glucose and sucrose, while white‐fleshed ones have higher fructose.^19^, ^43^ However, white‐fleshed fruits have a larger concentration of fructose (2.38 g 100 mL^−1^) and a better‐balanced ratio of glucose to fructose (2.13), which is more desirable as inverted sugar due to its sweetness; its potential for application in the industry should therefore not be disregarded.

**Figure 1:**
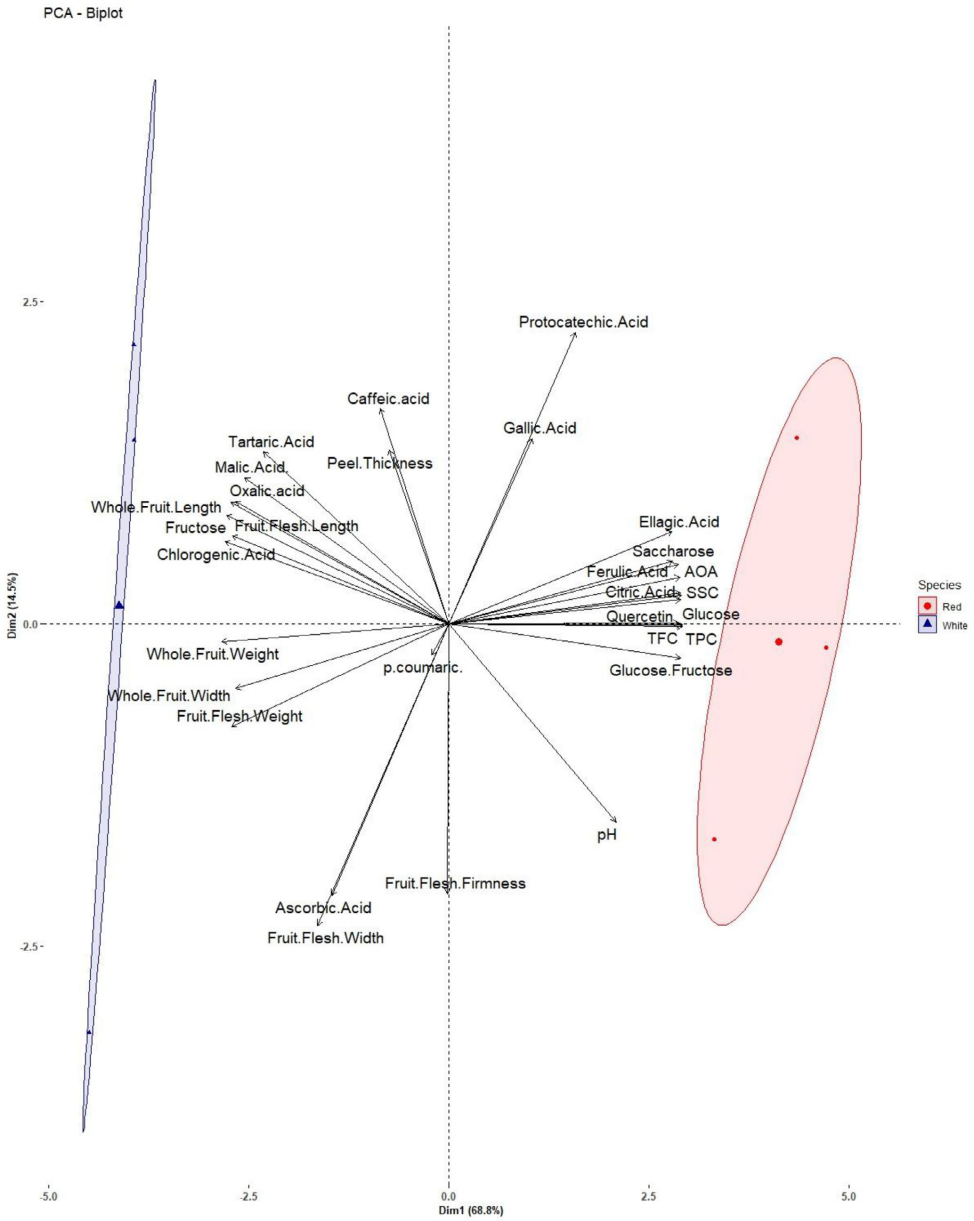
Bi‐plot distribution of the traits investigated across cultivars. TPC, total phenolic content; TFC, total flavonoid content; AOA, antioxidant activity; SSC, soluble solid content; Glucose.Fructose represents glucose/fructose ratio.

PCA was conducted to further explore the distribution of traits across different altitudes and varieties, with the findings presented in Fig. 1. This method has also been used in several adaptation and selection studies, including those on pitaya,^43^ strawberries,^44^ and rosehips.^45^ Main PCA components explained 83.3% of the total variation (PC1 68.8%, PC2 14.5%, respectively). The findings showed that pitaya varieties had varied characteristics since they were found on different planes. Therefore, the appropriate variety selection is crucial in achieving targeted goals for processing, breeding, or fresh consumption, as demonstrated. White‐fleshed pitayas are known for their large fruit size, making them suitable as parental varieties in breeding programs aimed at developing new genotypes with larger fruits. It is widely recognized that large‐fruited genotypes with a high ratio of soluble solid content to titratable acidity are a key breeding goal that is widely acknowledged for nearly all fruit species.^45^, ^46^ Similarly, red‐fleshed varieties might enhance the phytochemicals with the antioxidant effect of the next generation. Red‐fleshed varieties may also be used to produce fermented products such as vinegar and wine due to their rich flavonoids that give the products a bitter and astringent flavor.^47^ Additionally, red‐fleshed ones could be favored for fresh consumption due to their higher antioxidant activity. This antioxidative effect also increases the stability of processed products by suppressing reactive oxygen species, making them ideal for use as raw materials in industrial applications.

**Figure 2:**
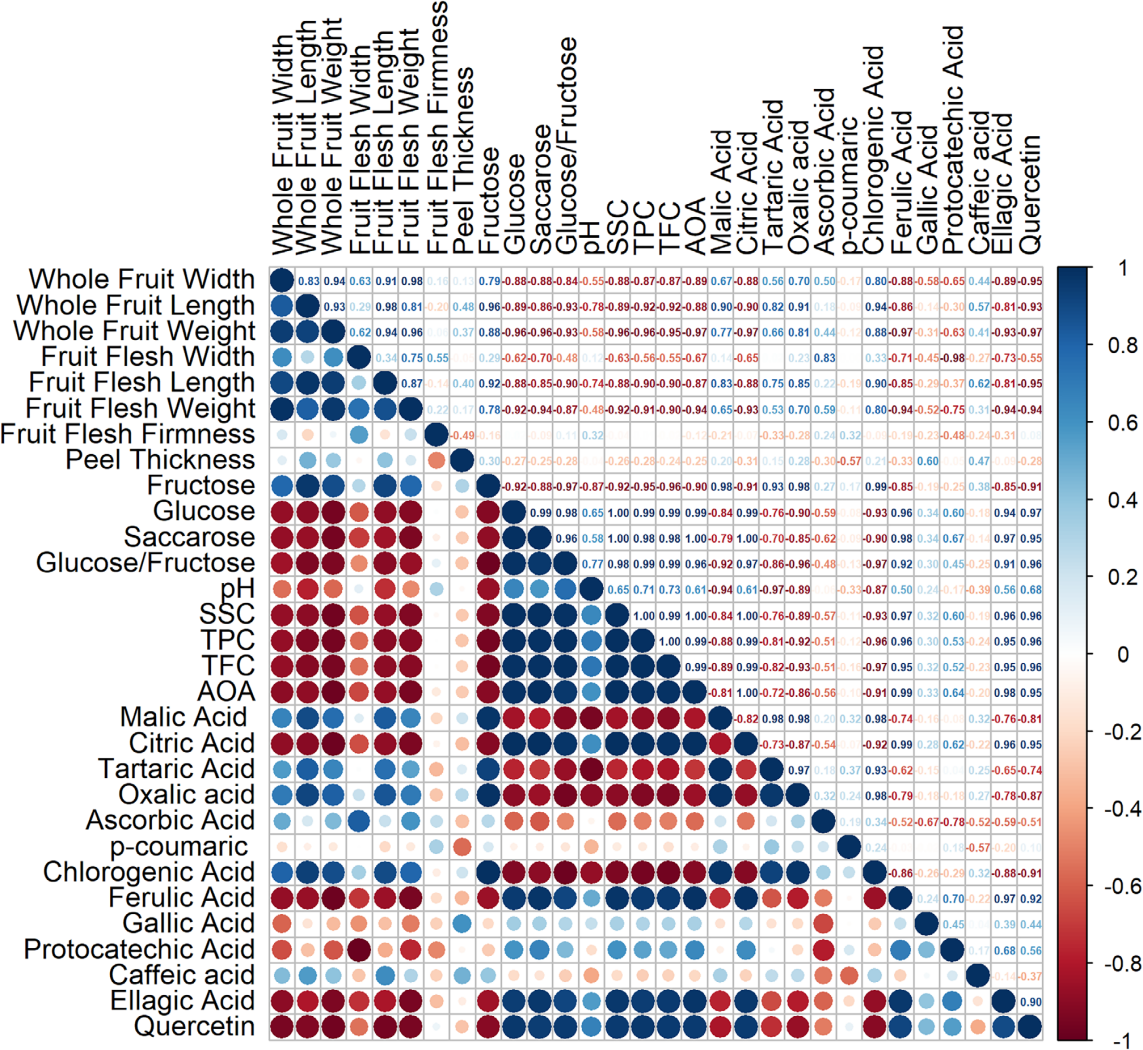
Correlations among the characteristics investigated. TPC, total phenolic content; TFC, total flavonoid content; AOA, antioxidant activity (DPPH assay); SSC, soluble solid content.

Understanding the relations between various characteristics contributes to reducing the time and expense of cultivation and breeding efforts. Figure 2 displays the correlation coefficients found among the traits that were investigated. Considering the whole fruit, positive strong correlations were revealed among fruit width and fruit length (*r* = 0.83). Simultaneous longitudinal and horizontal development occurs during the cell expansion stage, which explains why they have such a strong link. Fruit weight was shown to have high positive associations with fruit length and width, respectively, at *R* ^2^ = 0.62 and 0.94, indicating how fruit size directly influences weight. The literature supports our findings, confirming robust and affirmative correlations between fruit weight and size.^26^, ^48^, ^49^

The acidic environment in fruits not only limits the activity of microbes that cause spoilage but also allows for higher levels of accumulation of phenolic compounds with antioxidant effects.^25^, ^50^ The main contributors to creating an acidic environment – organic acids – were discovered to be inversely correlated with pH (Fig. 2). As expected, rising amounts of individual phenolics that are respected for their antioxidant effect increased the TPC, TFC, and antioxidant activity. All sugar forms examined were found to have essential effects on soluble solid content. Furthermore, the data demonstrated that the presence of sugar enhanced the accumulation of phenolics, supporting the sugar–phenolic interaction in the phenylpropanoid pathway.^51^ Although the major quantitative trait loci for these traits were found to co‐locate on the same linkage groups (LG1 and LG6) in plum,^52^ these compounds can be interconverted when necessary.^53^ As a result, outcomes may occasionally suggest a negative relationship between them.^48^ Negative correlations between certain traits are believed to result from this phenomenon. Studies conducted on different fruit species support the findings of the study.^25^, ^49^

**Figure 3:**
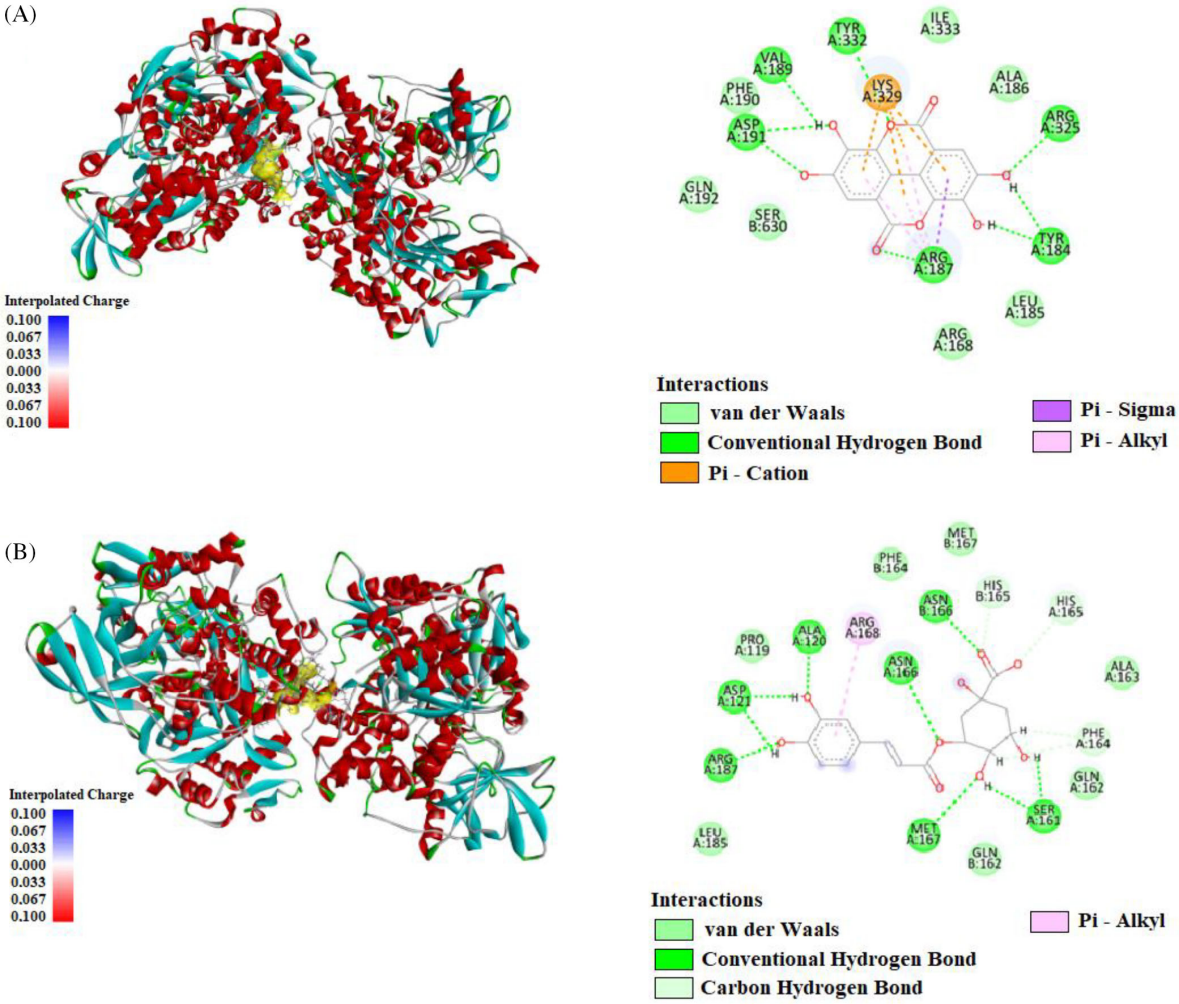
Molecular docking process of (A) ellagic acid and (B) chlorogenic acid with human cytosolic aconitase (2B3Y).

Molecular docking analyses reveal antioxidant compounds' binding energies and interactions with the human IRP1 protein. According to the results, ellagic acid exhibits the strongest interaction with the IRP1 protein, with a binding energy of −8.9 kcal moL^−1^, followed by chlorogenic acid at −7.8 kcal moL^−1^, ferulic acid at −5.8 kcal moL^−1^, and malic acid at −5.5 kcal moL^−1^ (Supporting Information, Table S1). Ellagic acid forms strong hydrogen bonds with amino acids, namely ARG187, ASP191, ARG325, and TYR332 in chain A of the IRP1 protein, and also engages in Pi–cation, Pi–alkyl, and Pi–sigma interactions with LYS329 and ARG187 (Fig. 3(A)). Chlorogenic acid binds through hydrogen bonds with various amino acids in chains A and B, including ALA120, ASN166, MET167, ARG187, ASP121, and SER161. Additionally, chlorogenic acid exhibits carbon–hydrogen bonds with PHE164 and SER161 as well as Pi–alkyl interactions with ARG168 (Fig. 3(B)).

**Figure 4:**
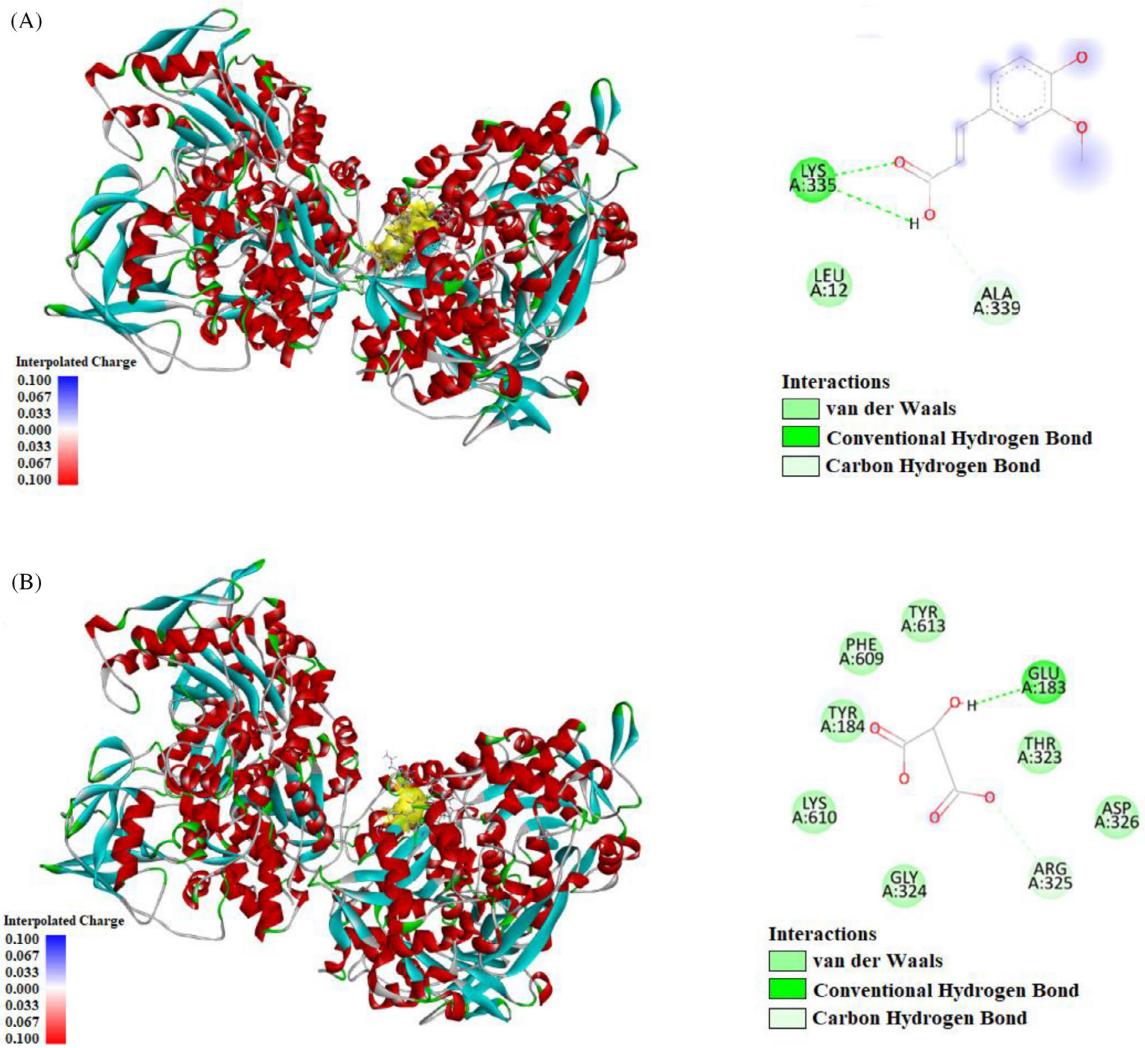
Molecular docking process of (A) ferulic acid and (B) malic acid with human cytosolic aconitase (2B3Y).

Ferulic acid interacts with LYS335 and ALA339 through hydrogen bonds (Fig. 4(A)), while malic acid forms hydrogen bonds with GLU183 and ARG325 (Fig. 4(B)). These compounds' binding energies and interactions may support their antioxidant properties, such as DPPH radical scavenging activity. The interaction of compounds like chlorogenic acid, ferulic acid, ellagic acid, and malic acid with the IRP1 protein, which plays a critical role in iron metabolism, could contribute to a better understanding of antioxidant mechanisms.

## CONCLUSIONS AND RECOMMENDATIONS

Pitaya represents a fascinating subject of scientific inquiry, offering a captivating blend of economic significance, nutritional richness, and potential health benefits. As research continues to unveil its myriad therapeutic properties, pitaya holds promise as a valuable dietary resource with the power to enhance human health and well‐being on a global scale.

In conclusion, a considerable variation was observed between pitaya species regarding their physicochemical characteristics. Among the species investigated, red‐fleshed pitaya fruits exhibited an exceptional nutritional profile and antioxidant activity, whereas white‐fleshed fruits came to the fore with their pomology. The outcome of multivariate analyses showed that pitaya species are valuable for various purposes, such as fresh consumption, breeding and vinegar. Correlation analysis revealed a coordinated accumulation of sugars and phenolics. Molecular docking simulations display how major compounds and IRP1 proteins, which play a critical role in iron metabolism, effectively interact to improve the antioxidant mechanisms.

## FUNDING INFOMATION

The publication of this article was funded by the Open Access Fund of Leibniz Universität Hannover.

## AUTHOR CONTRIBUTIONS

Kerem Mertoglu: conceptualization, investigation, formal analysis, data curation, methodology, writing – original draft, review and editing. Annik Fischer: Formal analysis, methodology, review and editing. Sina Zargarchi: formal analysis, data curation, methodology and writing – original draft. Melekber Sulusoglu Durul: conceptualization, review and editing. Magdalena Köpsel: formal analysis, data curation and methodology. Erdi Can Aytar: data curation and methodology. Barıs Kaki: data curation and methodology. Ibrahim Bulduk: data curation and methodology. Tuba Esatbeyoglu: conceptualization, methodology, supervision, funding acquisition, project administration, review and editing.

## CONFLICT OF INTEREST

The authors declare that they have no known competing financial interests or personal relationships that could have appeared to influence the work reported in this paper.

## Supporting information


**Data S1.** Supporting Information.

## Data Availability

The data that support the findings of this study are available from the corresponding author upon reasonable request.
