# Supplementary material for: Comparative analysis of pomological and phytochemical characteristics in white‐ and red‐fleshed pitaya ( Hylocereus spp.), with molecular docking insights into key bioactive compounds
Source: J Sci Food Agric. 2025 Nov 19;106(4):2200–10. doi: 10.1002/jsfa.70322 (PMC12872244; doi:10.1002/jsfa.70322)
Supplement: Supplementary file 1 — Data S1. Supporting Information. [file JSFA-106-2200-s001.docx]

**Supplementary Material Table 1.** Docking scores and report of predicted interactions of docked conformations of major compounds with 2BYL

| **Ligand** | **Protein** | **Binding Energy (kcal·moL^-^¹)** | **Amino acid** | **Interacting** | **Distance** |
| --- | --- | --- | --- | --- | --- |
| Chlorogenic acid | 2B3Y | -7.8 | A: ALA120:HN -: [001:O8 | Conventional Hydrogen Bond | 2.55 |
|  |  |  | A: ASN166:HN -: [001: O1 | Conventional Hydrogen Bond | 2.08 |
|  |  |  | A: MET167:HN -: [001: O4 | Conventional Hydrogen Bond | 2.16 |
|  |  |  | A: ARG187:HH21 -: [001: O9 | Conventional Hydrogen Bond | 1.73 |
|  |  |  | B: ASN166:HN -: [001: O6 | Conventional Hydrogen Bond | 1.79 |
|  |  |  | : [001:H2 - A: SER161:O | Conventional Hydrogen Bond | 1.64 |
|  |  |  | : [001:H3 - A: SER161:O | Conventional Hydrogen Bond | 1.85 |
|  |  |  | : [001:H12 - A: ASP121:OD1 | Conventional Hydrogen Bond | 1.95 |
|  |  |  | : [001:H13 - A: ASP121:OD1 | Conventional Hydrogen Bond | 1.87 |
|  |  |  | A: HIS165:HD2 -: [001: O5 | Carbon Hydrogen Bond | 2.64 |
|  |  |  | A: ASN166: HA -: [001: O1 | Carbon Hydrogen Bond | 2.91 |
|  |  |  | B: HIS165: HA -: [001: O6 | Carbon Hydrogen Bond | 1.68 |
|  |  |  | : [001:H4 - A: PHE164:O | Carbon Hydrogen Bond | 2.58 |
|  |  |  | : [001:H5 - A: SER161:O | Carbon Hydrogen Bond | 2.88 |
|  |  |  | : [001:H5 - A: PHE164:O | Carbon Hydrogen Bond | 2.32 |
|  |  |  | : [001 - A: ARG168 | Pi-Alkyl | 4.20 |
| Ferulic Acid | 2B3Y | -5.8 | A: LYS335:HZ2 - :4451: O4 | Conventional Hydrogen Bond | 3.05 |
|  |  |  | :4451:H10 - A: LYS335:O | Conventional Hydrogen Bond | 2.07 |
|  |  |  | A: ALA339: HA - :4451: O3 | Carbon Hydrogen Bond | 2.22 |
| Ellagic Acid | 2B3Y | -8.9 | A: ARG187:HH22 -: [001: O8 | Conventional Hydrogen Bond | 2.06 |
|  |  |  | A:ASP191:HN -: [001: O2 | Conventional Hydrogen Bond | 2.72 |
|  |  |  | A:ASP191:HN -: [001:O3 | Conventional Hydrogen Bond | 2.03 |
|  |  |  | A: ARG325: HE -: [001: O6 | Conventional Hydrogen Bond | 1.90 |
|  |  |  | A: TYR332: HH -: [001: O4 | Conventional Hydrogen Bond | 1.65 |
|  |  |  | : [001:H2 - A: VAL189:O | Conventional Hydrogen Bond | 3.99 |
|  |  |  | : [001:H3 - A: TYR184:O | Conventional Hydrogen Bond | 4.05 |
|  |  |  | : [001:H4 - A: TYR184:O | Conventional Hydrogen Bond | 2.87 |
|  |  |  | A: LYS329:NZ -: [001 | Pi-Cation | 2.52 |
|  |  |  | A: LYS329:NZ -: [001 | Pi-Cation | 2.94 |
|  |  |  | A: LYS329:HZ1 -: [001 | Pi-Cation; Pi-Donor Hydrogen Bond | 3.68 |
|  |  |  | A: LYS329:HZ1 -: [001 | Pi-Cation; Pi-Donor Hydrogen Bond | 3.52 |
|  |  |  | A: ARG187: HA -: [001 | Pi-Sigma | 4.50 |
|  |  |  | : [001 - A: ARG187 | Pi-Alkyl | 3.67 |
|  |  |  | : [001 - A: ARG187 | Pi-Alkyl | 3.05 |
|  |  |  | : [001 - A: LYS329 | Pi-Alkyl | 2.07 |
|  |  |  | : [001 - A: ARG187 | Pi-Alkyl | 2.22 |
| Malic acid | 2B3Y | -5.5 | : [001:H6 - A: GLU183:O | Conventional Hydrogen Bond | 1.62 |
|  |  |  | A: ARG325: HA -:[001:O2 | Carbon Hydrogen Bond | 2.36 |
